# The effect of sleeping posture on occupant injury in frontal coach crashes

**DOI:** 10.1038/s41598-025-86670-z

**Published:** 2025-03-27

**Authors:** Sun Tingting, Zaidi Mohd Ripin, Chan Ping Yi, Mohamad Ikhwan Zaini Ridzwan

**Affiliations:** 1 Neurorehabilitation Engineering and Assistance Systems Research (NEAR), School of Mechanical Engineering, Engineering Campus, Universiti Sains Malaysia, 14300 Nibong Tebal, Penang Malaysia; 2 Yancheng Institute of Technology, Xiwangdadao, Tinghu District, Yancheng, 224000 Jiangsu China; 3 School of Engineering, Monash University Malaysia, Jalan Lagoon Selatan, Bandar Sunway, 47500 Subang Jayam, Selangor Malaysia

**Keywords:** Sleeping posture, Frontal coach crash, Occupant injury, Mechanical engineering, Software

## Abstract

The safety of coaches in frontal crashes is attracting attention in various countries. Coach crash studies are done based on standard postures. Occupants travelling long distances in coaches may fall asleep, with postures that can be different from the standard posture. This paper investigates the effects of different sleeping postures on occupant injuries in frontal coach collisions by using sled test model and dummies. Four different sleeping postures are considered including head tilted sideway, slide down on seat in neutral position, turned torso-head diagonal with backrest and turned torso-head perpendicular with backrest posture. The results showed that the head tilted sideway posture increases occupant head injuries with the Head Injury Criteria (HIC) values of 399.3 to 494.6 and the peak head acceleration over 3 milliseconds (H3ms) reaches a maximum of 88.53 g which is above the accepted threshold of 80 g. Slide down on seat in neutral position posture reduces occupant head injuries where the HIC decreased by 28.47%, and for H3ms the decrease is 12.15%. Turned torso posture reduces neck injuries, and all those four sleeping postures are beneficial to reduce thorax injuries. The different turned torso postures simultaneously include the lowest risk and the highest risk postures.

## Introduction

People in various countries favor coaches as a means of public transport, especially for long distances. In 2019, Poland transported a total of 459.9 million occupants^1^. However, Enhanced Coach and Bus Occupant Safety (ECBOS) project found that frontal crashes account for 70% of coach accidents^2^. Frontal crashes of coaches can lead to many casualties. This has caused widespread concern in society and the government. In the Netherlands, 83.3% of all traffic fatalities are caused by head-on coach collisions^3^. The 2020 Traffic Safety Facts Annual Report of NHTSA states that frontal coach collisions rank first causing 55.56% of all fatalities and 51.43% of all injuries^4^.

To protect the safety of occupants, various countries are introducing regulations on frontal crashes of coaches. Researches on frontal crashes of coaches mainly refer to ECE R80 (United Nations Economic Commission for Europe Regulation No. 80)^5^ and FMVSS 208 (U.S. Federal Motor Vehicle Safety Standard 208)^6^ which specify experimental conditions, dummy sitting postures, and injury criteria. These regulations formed the evaluation basis of the effects of coach interior structure and seat parameters on occupant injury^7–9^. In addition, there are specifications for the head, neck, chest, and lower extremity injury limit values for test dummies to evaluate occupant safety.

The standard posture for dummies in the experiment is required to be with the back against the seat back, the head at center position and the hands in the lap. However, occupants would normally choose to sit in a comfortable posture when travelling in coaches for long periods of time. Evidence of recorded the values of the angles of the neck, lumbar, and knee of nine volunteers a comfortable posture showed angles which differed significantly from the standard posture^10^. The angles of the femur-tibia and back-femur in the standard posture of the occupant are about 90° and 100°, respectively, while the corresponding maximum angles in the volunteer’s comfortable sitting posture are 127° and 120°. The difference between standard experimental and actual sitting posture suggests that the experimental results of injury values may not be representative. As a result, some studies have begun to focus on the impact of non-standard postures and the effect of posture changes in different body parts in a passenger car on occupant injuries^11^. The results revealed that alterations in one body part exacerbated injuries in other parts. For example, placing the left elbow on the center console resulted in an increase of the left humerus proximal resultant moment from 13 Nm to 22 Nm, as well as a 10 mmincrease in sternum excursion. Varying the occupants’ postures randomly resulted in variations in injury outcomes with the Head Injury Criterion More worryingly is the data where although only (HIC) for the 95th percentile male dummy experienced the variation from − 11% to + 104%^12^. The HIC is defined as the head resultant acceleration over a specified time interval, and can be calculated from the head acceleration^13^. More worryingly is the data where although only a quarter of vehicles are on the road at night, the likelihood of severe injury or death is greater during nighttime driving compared to daytime driving^14^. Severe and fatal injuries caused by nighttime driving range from 42.9 to 82.7%, compared to 20.9–25.1% caused by daytime driving^15^. Therefore, the impact of the posture chosen by occupants during nighttime travel-specifically sleeping postures-on coach collisions is also an aspect that requires our attention.

Previous research on frontal collisions in coaches primarily focused on changes in the angles of the occupant’s legs, back and neck based on deviations from the standard seating posture. However, when passengers are in a sleeping posture, their posture will differ from the standard posture in the experiment and previous studies on seating postures of passengers. In the case of sleeping postures, the occupant’s entire body leans against the backrest, and may even involve a rotation of the torso angle. This could lead to injuries during a real frontal collision that are different from those observed. Additionally, the variations in sleeping postures may cause varying degrees of impact on different parts of the body. Therefore, this paper selects different sleeping postures to study their effects on passenger injuries.

## Method

A series of simulations with different sleeping postures of occupants in coaches were performed by sled test. It includes four different sleeping postures and variations in angles within the same posture, 12 simulations in total.

### Models

**Fig. 1:**
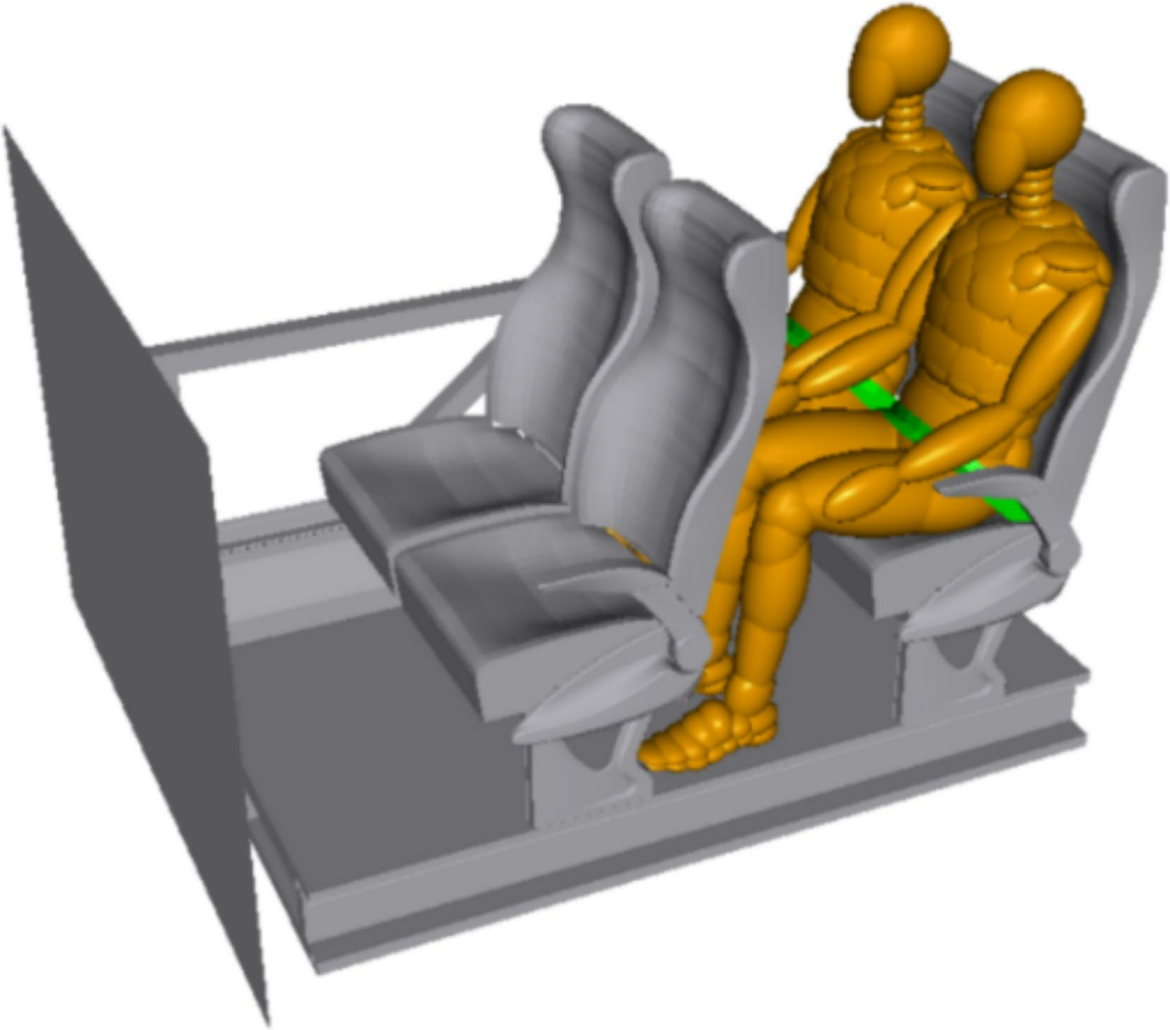
Sled test model sitting two 50th percentile male dummies with two-point seat belts.

The method of coupling MADYMO and LS-DYNA is used to simulate the frontal collision of a dummy in a sled model. The MADYMO version used is MADYMO 75, and the LS-DYNA version is LS-DYNA R11. The sled finite element model is used to simulate the internal structure of the coach, including the floor, side structure and two rows of seats. Following the requirements of the sled test in ECE R80 ^5^, two 50th percentile male dummies with two-point seat belts are placed in the rear seats. Most countries require that, except for the first-row passengers and the driver, all other passengers in a coach be equipped with two-point seat belts, and the use of these seat belts is mandatory. For example, the Chinese standard regulation-GB7258 ^16^. The distance between the two rows of seats is 750 mm. The dummies sit upright with their feet flat on the floor and their heads not in contact with the backrest. In MADYMO, the dummies are used Hybrid III 50th percentile dummies in the MADYMO, and are invoked and coupled with the sled model, as shown in Fig. 1.

**Fig. 2:**
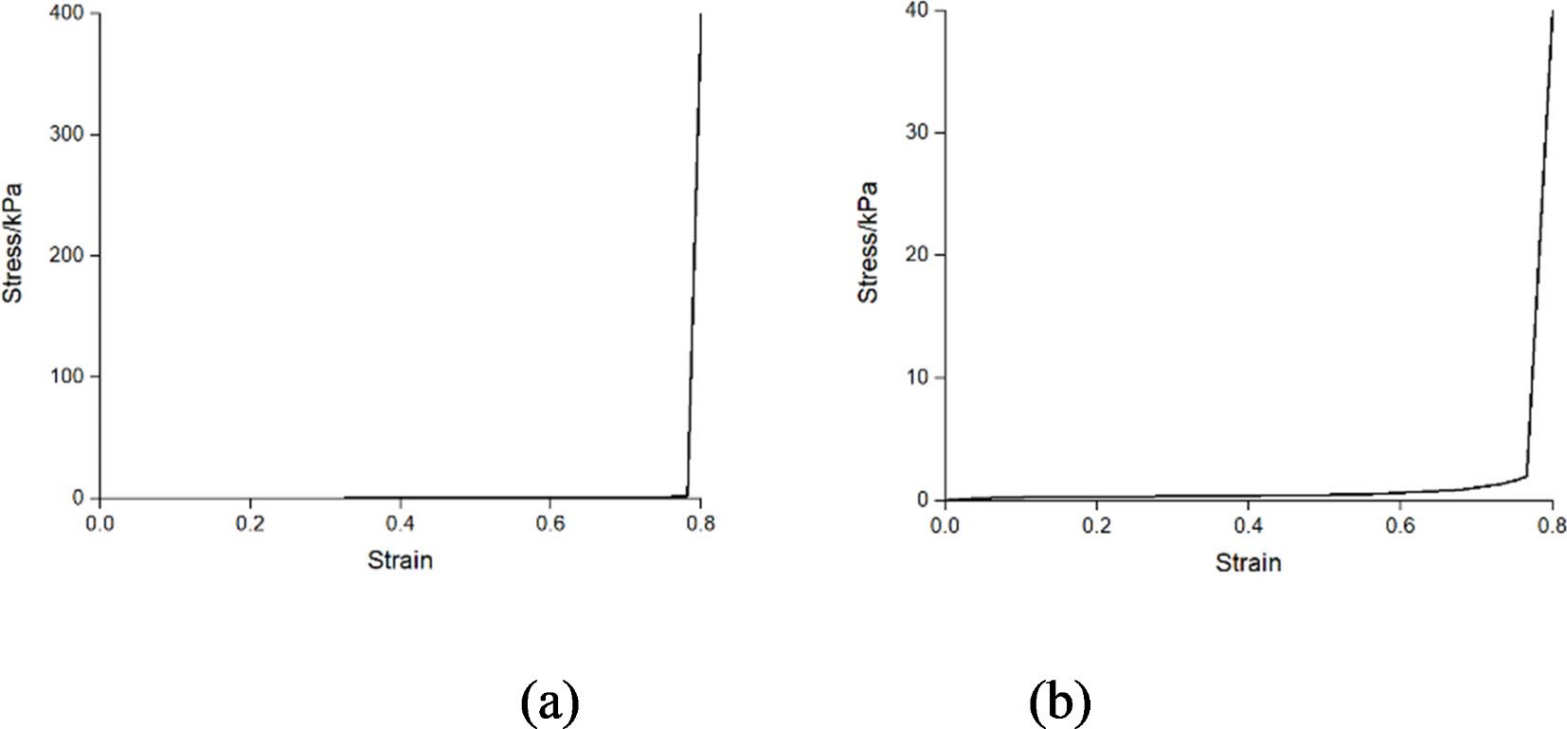
The stress-strain curves of different parts of seat: (**a**) seat cushion, (**b**) backrest.

Most of the sled finite element model are constructed in steel, while the seat cushions and backrests are made of foam materials. The materials of the seat cushion and the seat backrest are defined using nonlinear material properties, and the stress-strain curves are shown in Fig. 2. When the dummy contacts the front seat, the foam of the seat backrest will deform due to the applied impact force. This deformation helps to some extent in cushioning the impact and reducing the injury to the dummy’s head. Seat belts combine finite element with multi-rigid-body. The contact position with the dummy is represented by the finite element, which can simulate the relative sliding between the dummy and the seat belt. The finite element segment of the seatbelt is 767 mm in length and 50 mm in width. The length of the multi-rigid body seatbelt at both ends is 10 mm. When combining the dummy with the sled model, contact is defined between the dummy and the seat, with the coefficient of friction set to 0.5.

**Fig. 3:**
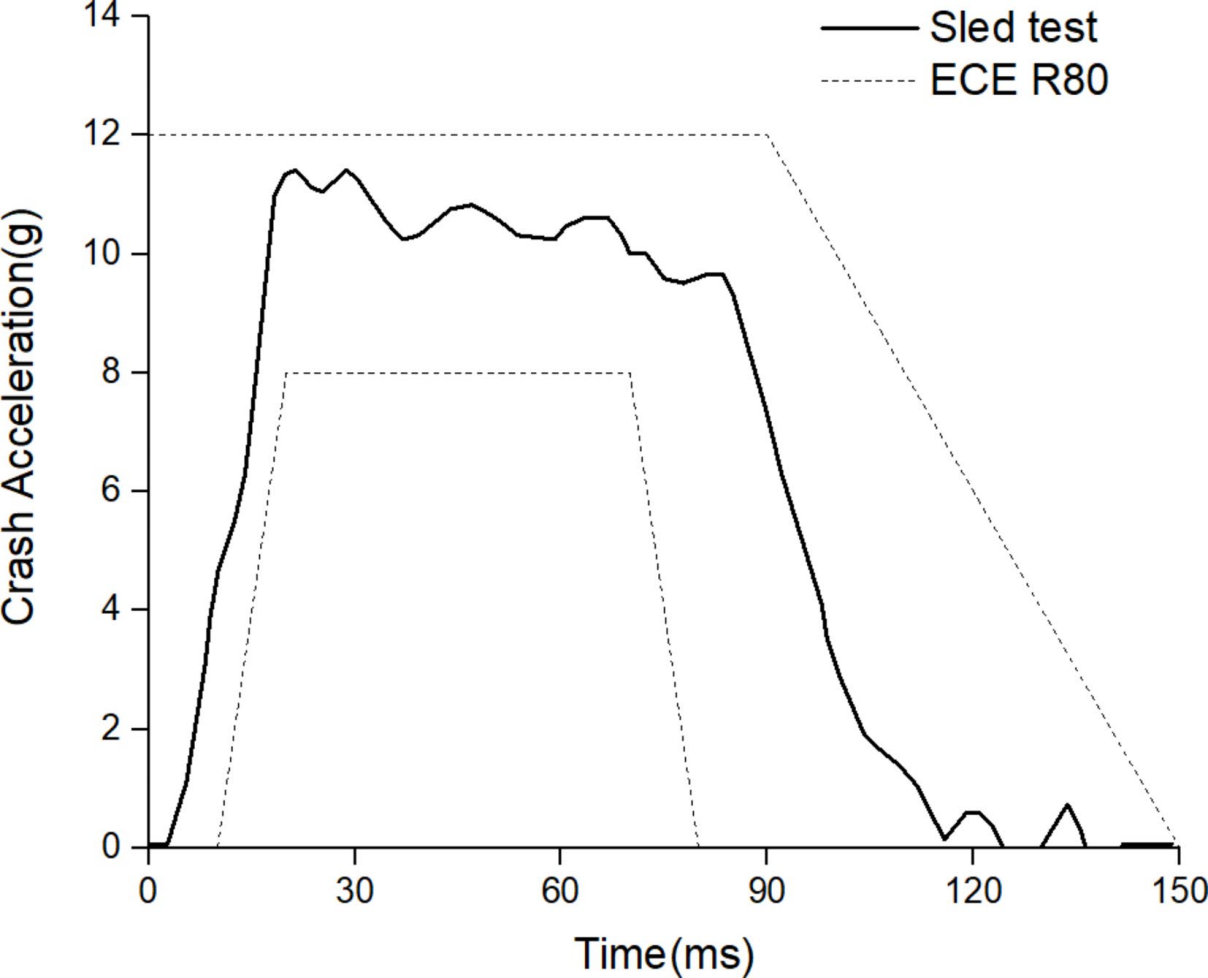
The crash acceleration curve in accordance with ECE R80^8^.

The collision velocity is 30 km/h, and the collision acceleration curve conforms to the ECE R80 corridor as shown in Fig. 3^8^. The sled model consists of approximately 280,000 elements, and the duration of each crash simulation is 180ms. The joint of MADYMO and LS-DYNA simulate frontal crashes of coaches and obtain injury values for dummies.

The sled model is verified by comparing the occupant injury values obtained in the simulation and actual sled test. The injury values of sled test and simulation for the occupant near the window are shown in Table 1. As seen in the Table 1, the errors for HIC, H3ms (head acceleration over 3 ms), My (extension moment of neck), ThAC (Thorax Acceptability Criterion), and FFC (Femur Force Criterion) are within 15%, which is in accordance with the requirements^17^.

**Table 1:** The comparison of occupant injury values between sled test and simulation.

Injury	HIC	H3ms	M_y_	ThPC	FFC	
					Left	Right
Sled Test	378	70.78 g	71.8Nm	22.02 g	0.80kN	1.59kN
Simulation	399.3	80.27 g	62.3Nm	20.31 g	0.71kN	1.42kN
Discrepancy	5.6%	13.4%	13.2%	8.4%	12.7%	12.0%

### Occupant sleeping postures

**Fig. 4:**
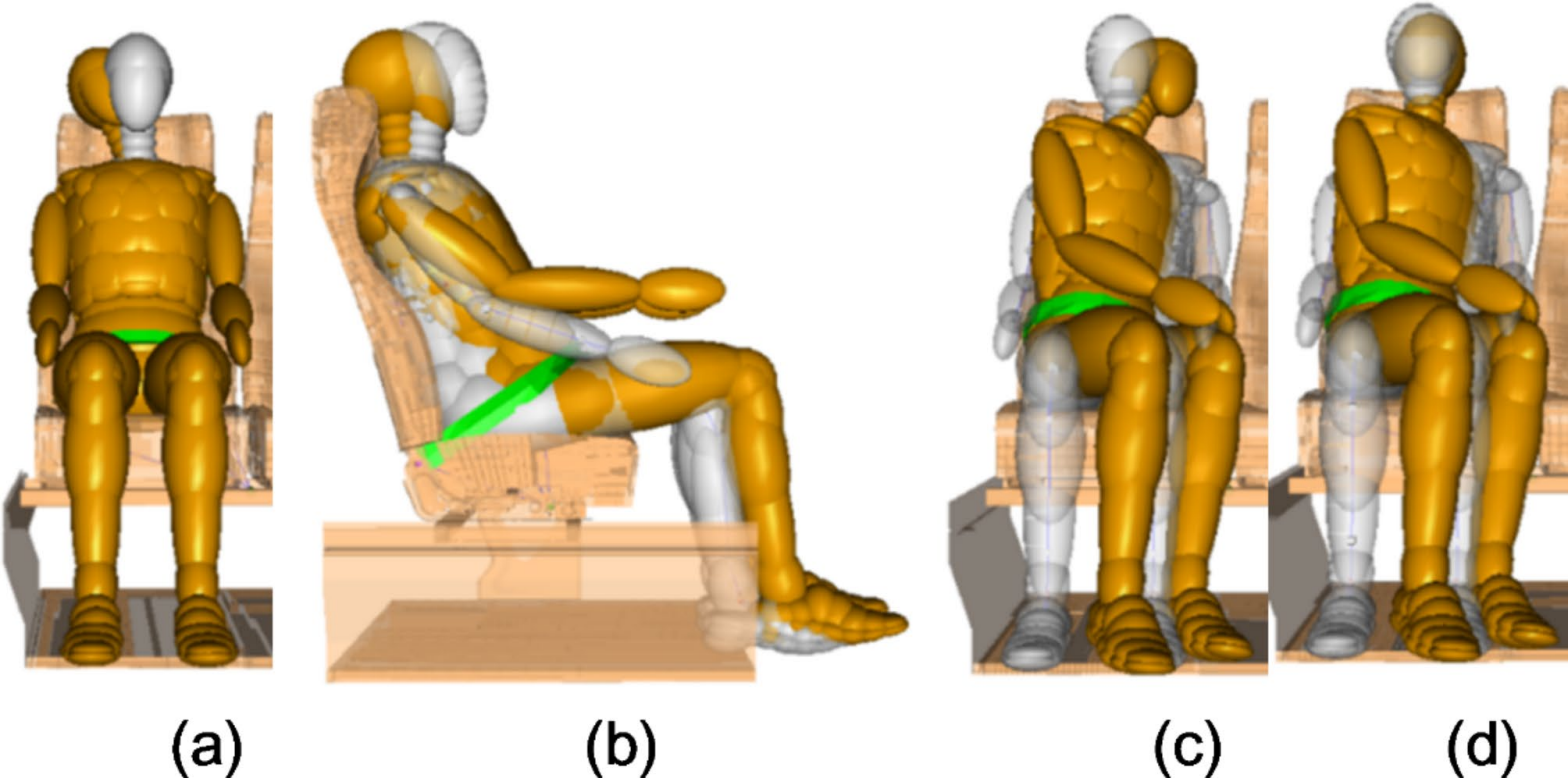
Different sleeping postures of occupants on the coach in color yellow (white dummy is the standard posture). (**a**) Head tilted sideway with α of 20°; (**b**) slide down on seat in neutral position β of 15°; (**c**) turned torso-head diagonal with backrest with γ of 60°; (d) turned torso-head perpendicular with backrest γ of 60°.

This study investigates the safety of occupants at different angles in each sleeping posture. In practice, occupants in coaches sleep in different postures. But there is no study in the literatures on the sleeping postures of occupants in coaches. Due to the similarity in interior space between airplanes and coaches, the sleeping postures of occupants in economy class aircraft were used to study the safety of occupants in different sleeping postures in coaches according to the research of Tan et al.^18^. Four different sleeping postures were selected based on the statistics in the literature. These postures are head tilted sideway posture, slide down on seat in neutral position posture, turned torso-diagonal with backrest posture, and turned torso-head perpendicular with backrest posture. A schematic diagram of the sleeping postures is shown in Fig. 4.

The head of occupant with head tilted sideway posture is deflected to the left or right around the X-axis. The lumbar of occupant with slide down on seat in neutral position posture rotates around the Y-axis with the hips to move forward. In the turned torso-head diagonal with backrest posture, the occupant turns hips around the Z-axis, and the head also rotates along the hips, diagonal with backrest. The occupant in the condition of turned torso-head perpendicular with backrest turns the hips around the Z-axis, and the head remains facing the front seat, perpendicular to the backrest.

**Fig. 5:**
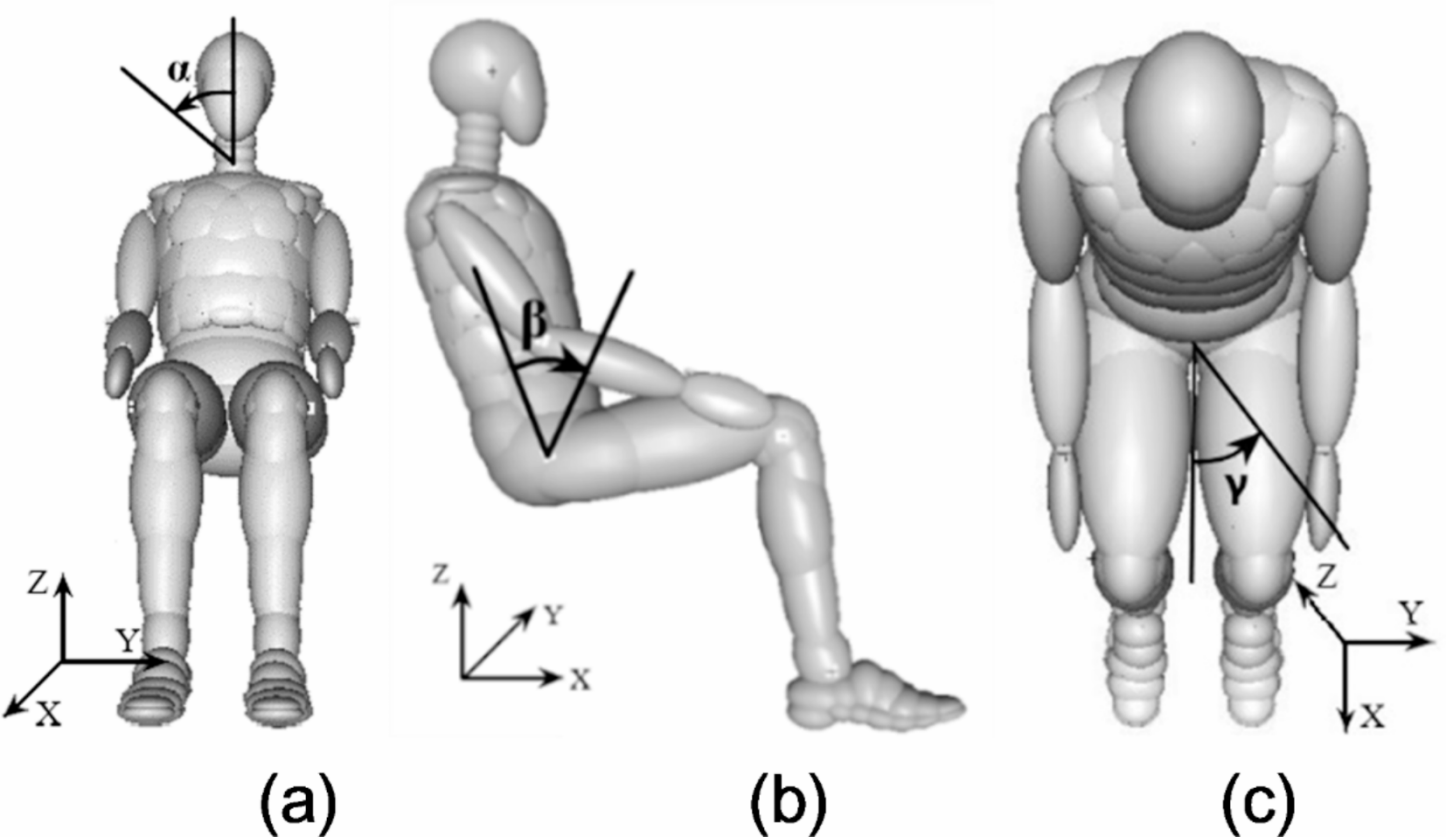
Schematic diagram of angle definition of (**a**) α, (**b**) β, (**c**) γ.

The definition of different angles for dummy is illustrated in Fig. 5. The positive directions of coordinate system are defined as X-axis to the front of the dummy, Y-axis to the left of the dummy and Z-axis upwards. The definitions of three angles are as follows. α is the angle of rotation of the head from standard posture around the X-axis; β is angle where the lumbar is turned from standard posture around the Y-axis; γ is angle of rotation of the hips from a standard posture around the Z-axis. In these angles, the positive direction of rotation is clockwise.

There are three angles (α, β and γ) and the specific angle settings are shown in Table 2. Here, α takes the positive direction, while β and γ take the opposite direction. For convenience, the positive and negative signs are not used in the table. The limit values of α, β and γ are chosen based on the fact that the dummy body parts could not traverse the seat and turned torso do not exceed 90°. In MADYMO the dummy’s H-point position is adjusted to ensure that the dummy’s head is resting on the backrest and the body is in contact with the backrest. Sometimes it is also necessary to adjust the position of the arms and femurs so that they do not interfere with the seat and the other dummy.

**Table 2:** Settings of angles in different sleeping posture.

No.	Postures		Parameters (°)			
Posture 1	Head tilted sideway		α	0	10	20
Posture 2	Slide down on seat in neutral position		β	5	10	15
Posture 3	Turned torso	head diagonal with backrest	γ	30	60	90
Posture 4		head perpendicular with backrest				

### Occupant injury criteria

This paper combines the criteria in ECE R80 and ECE R94 (United Nations Economic Commission for Europe Regulation 94)^19^. ECE R80 is the regulation on the stability of seats in coaches and ECE R94 is the regulation on the safety of occupants in frontal crashes of M1 vehicles with a seating capacity of up to 9 seats. The body parts include the head, neck, thorax, and femur as shown in Table 3. ECE R80 has only HIC, ThAC and FFC, which cannot comprehensively evaluate occupant injuries, so the criteria of H3ms, My, TI (Tibia Index), and knee sliding displacement in ECE R94 are added.

**Table 3:** The injury criteria and threshold.

Injury Criteria	HIC	H3ms	M_y_	ThPC	FFC	TI	Knee sliding displacement
Limit values	500	80 g	57Nm	30 g	10kN	1.3	15 mm

## Result

Twelve simulations were conducted for four sleeping postures and different angles for each posture. The standard posture was used as a reference to compare with the results of the different sleeping postures.

### Effect of head tilted sideway posture on occupant injuries

**Fig. 6:**
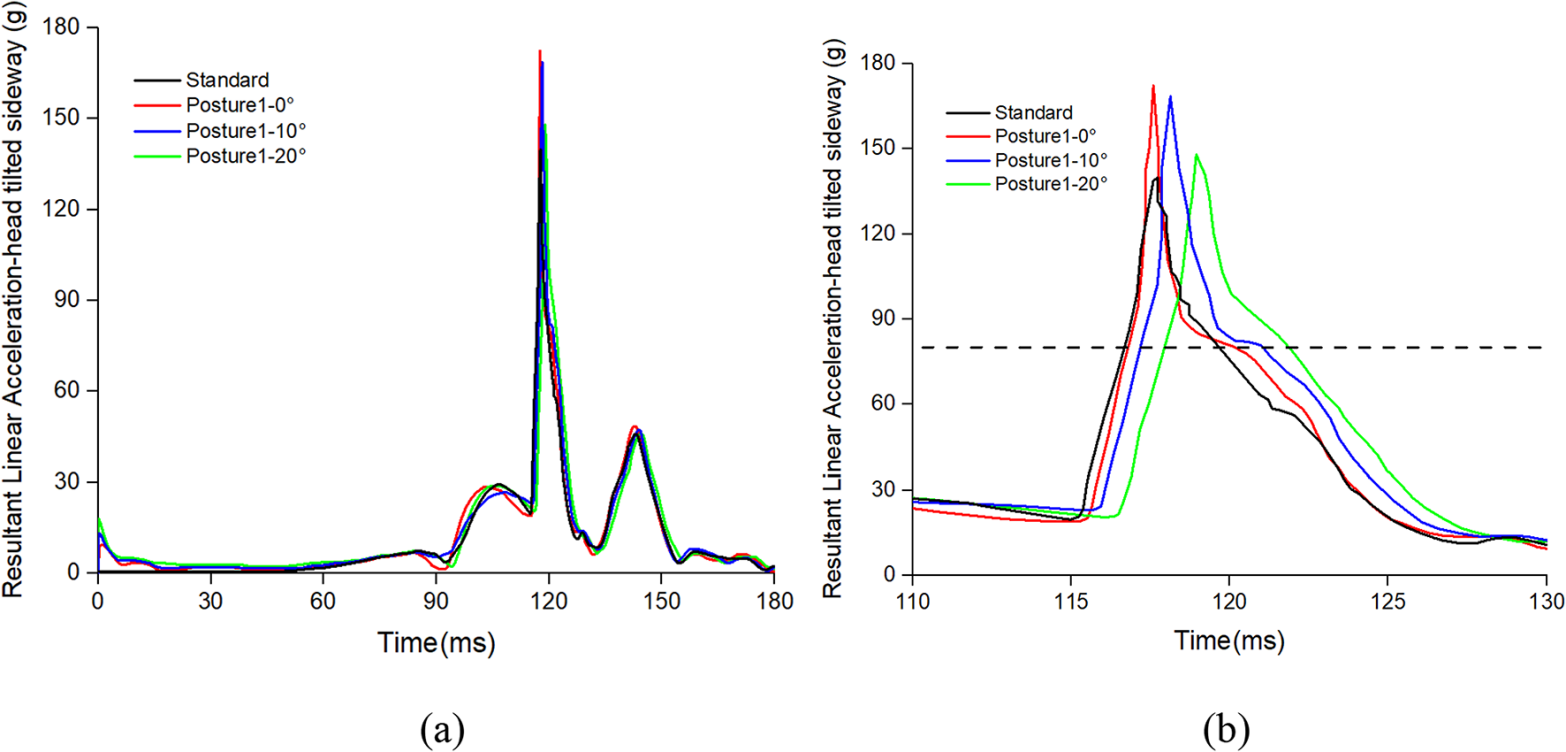
(**a**) The resultant linear acceleration and (**b**) the enlarged figure from 110-130ms of different head tilted sideway posture.

In the head tilted sideway posture, occupants move forward while their heads rotate from a tilted position towards the center during the collision process. Taking the Table 4 as an example with α of 20°, the time for the head to return to the central position is about 45ms. When the α is 10°, the time is approximately 35ms. Subsequently, the occupant continues to move forward until the knees impact the front seat. Then, the occupant’s head strikes the backrest of the front seat. In the standard posture, the time when occupants contact the front seats is around 117ms. But in the head tilted sideway posture, the contact time is between 117ms to 120ms. And the larger the angle, the later the contact time. At the moment the head impacts the front seat, the occupant’s head linear acceleration reaches its maximum. As shown in Fig. 6a, the peak acceleration is highest when α is 10°, reaching 172 g. As shown in Fig. 6b, the head acceleration peaks, ranked from highest to lowest, are at α of 0°, 10°, 20°, and the standard posture. After that, the dummy starts to rebound. All the angles are at around 150ms. There is also a slight difference in the deflection of the head in impact with the front seat. At the time of head impact with the front seat, the maximum differences in angular displacement values of the occupant at different angles, compared to the standard posture, are approximately 1.72°, 365.57°, and 2.87°.

**Table 4:**
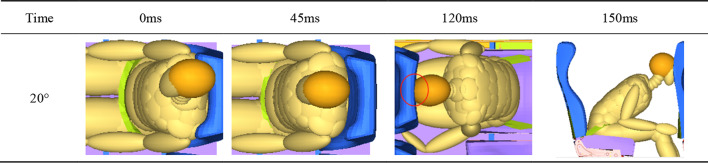
The dynamic responses of head tilted sideway posture with α of 20° in different time.

**Fig. 7:**
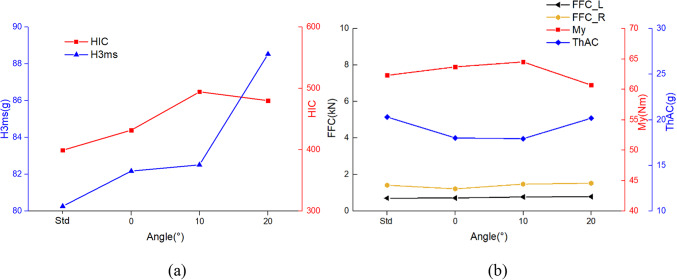
The comparison of occupant injuries for (**a**) head, (**b**) neck, thorax and femur between different head tilted sideway posture.

Occupant injury values under head tilted sideway posture are shown in Fig. 7. Head tilted sideway posture has a great effect on the occupant’s head. The values of HIC and H3ms of the occupants exceeded the values of the standard posture. Especially the HIC of the dummy increased from 399.3 in the standard posture to 494.6 (23.87%) with α of 10°. The H3ms rises with the increase in head deviation angle, reaching a maximum of 88.53 g (10.29%). The lower body of the occupant do not change under head tilted sideway posture, but as the angle increases, the value of FFC increases gradually, with a maximum increase of 11.27%. This is because the delayed time of the head impact on the front seats results in the femurs bearing the force of the impact for a longer duration. My varies less, between 60.7Nm and 64.5Nm. Head tilted sideway posture reduces injuries to the occupant’s thorax, and the values of ThAC at different angles are all lower than the ThAC in the standard posture.

### Effect of slide down on seat in neutral position posture on occupant injuries

As β increases, the distance between the knees and the front seat gradually decreases. Therefore, during the collision process in the coach, occupant’s hips move forward, and the occupant’s knee encounters the front seat. As shown in Table 5, the larger the β, the earlier the impact time. The collision time is advanced from 65ms in the standard posture to 40ms when β is 15°. Subsequently, the occupant moves forward around the lumbar until the head impacts the front seat. Impact time of the head with the front seat is between 115ms and 125ms, and it is delayed as the β increases. The occupant rebound phase occurs at 150ms. As shown in Table 5, there is a difference in the head impact position, and significant neck deformation is observed in the occupant. The location of the dummy’s head impact increases in height as the angle increases. At this moment, as the angle increases, the neck deformation shows a significant difference compared to that of the left-side passenger in the standard posture. It is also noted that when β is 15°, the occupant’s knees are still in contact with the front seat.

**Table 5:**
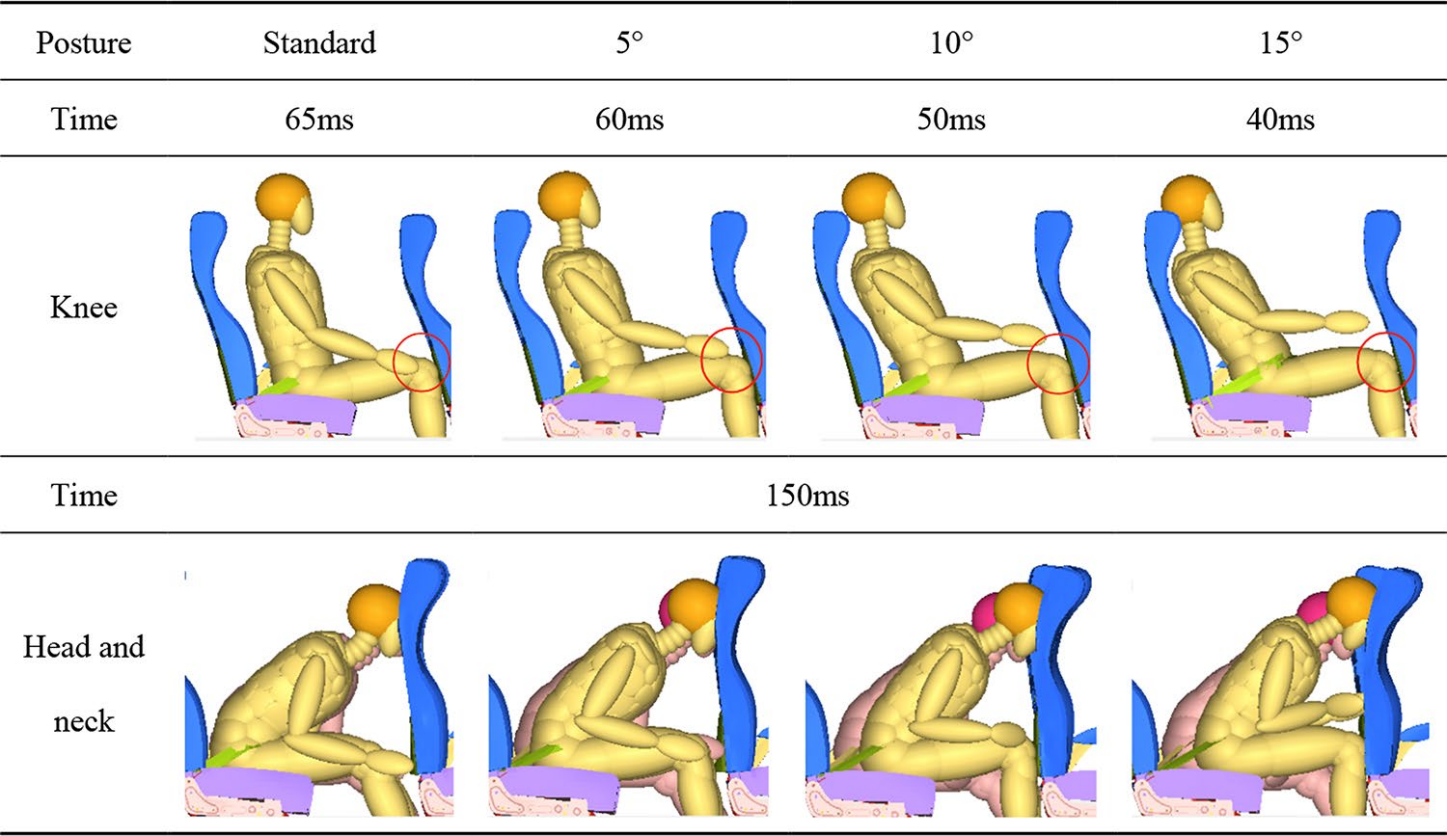
Schematic diagram of the time of collision between the occupant’s knees and the front seat at different angles of slide down on seat in neutral position posture.

Note: There are pink dummies in the seats on the left side in the row for ‘head and neck’.

**Fig. 8:**
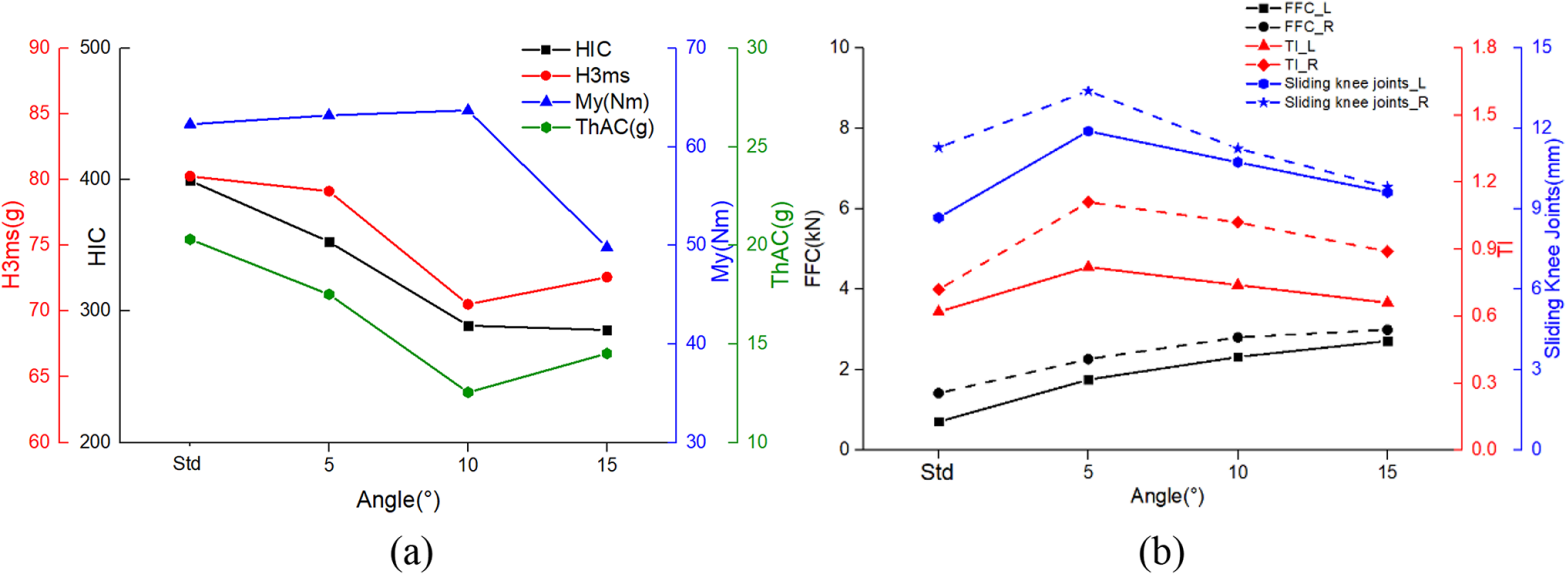
The comparison of occupant injuries for (**a**) head, neck, thorax, (**b**) femur, tibia and knee between different slide down on seat in neutral position posture.

The duration of the collision between the knees and the front seat became longer, which also cushioned the head impact force. As a result, both HIC and H3ms are lower than in the standard posture, as shown in Fig. 8. HIC reduced by a maximum of 28.47%, and H3ms reduced by up to 12.15%. My of neck criterion is lowest at the angle of 15°, measuring 49.8Nm. The rest exceeded the limit by 57Nm. Under the slide down on seat in neutral position posture, the thorax injury values are smaller compared to those under the standard posture. The increase contact time in legs with the seat leads to an increase in the FFC of the left femur from 0.71kN to 2.72kN, and the FFC of the right femur from 1.42kN to a maximum of 3.00kN. Meanwhile, the femur bears a larger axial force, thus increasing β reduced the injury to the tibia and the slippage of the knee. The maximum TI was 0.82 (left) and 1.11 (right), with maximum knee sliding displacement values of 11.90 mm (left) and 13.40 mm (right).

### Effect of turned Torso posture on occupant injuries

Turned torso posture includes head diagonal with backrest and head perpendicular with backrest. The dummy’s head in head perpendicular with backrest posture will return to the center firstly in a collision, compared to the posture of head diagonal with backrest. Then the dummy moves forward, with one lower limb colliding with the front seat before the other during a collision, as shown in Table 6. The right lower limb impacts with the foam area of the front seat, while the left lower limb impacts with the seat frame. Next, the occupant’s head impacts the front seat. In the two turned torso postures, the time for the right knee to impact the front seat is between 40ms and 50ms, while the time for the left tibia is approximately between 75ms and 95ms. Additionally, as the angle increases, the impact time of the right knee occurs earlier, and the impact time of the left tibia is delayed.

**Table 6:**
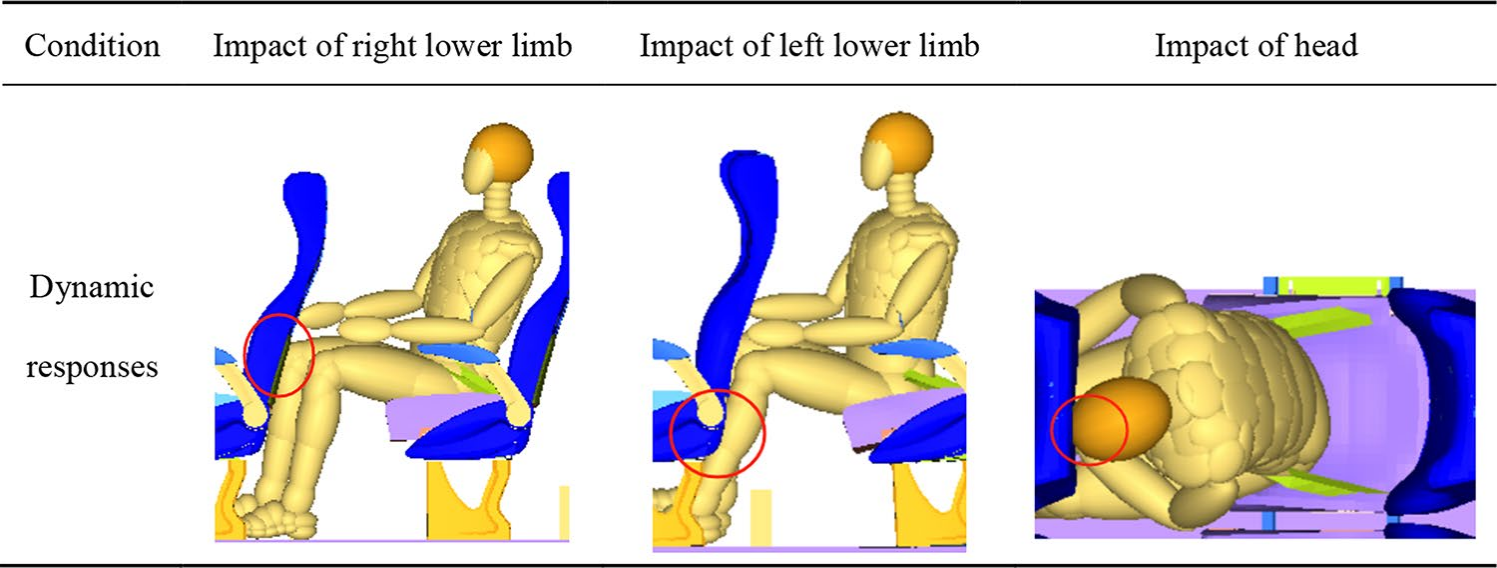
The dynamic responses in different impact body parts of turned torso posture with γ of 30°.

The occupant’s head collision time with the front seat is between 110ms and 115ms in turned torso posture, except when γ is 30° of head perpendicular with backrest, in which case the impact occurs between 115ms and 120ms. When the angle of γ increases, the collision time is earlier. The position of the occupant’s head relative to the front seat differ due to the varying initial head deflection angles in the two postures, as shown in Table 7, taking the 30° as an example. Under these two different turned torso sleeping postures, the head deflection angles differ by 1.15° around the X-axis, 1.72° around the Y-axis, and 5.73° around the Z-axis at the time of collision with the front seats.

**Table 7:**
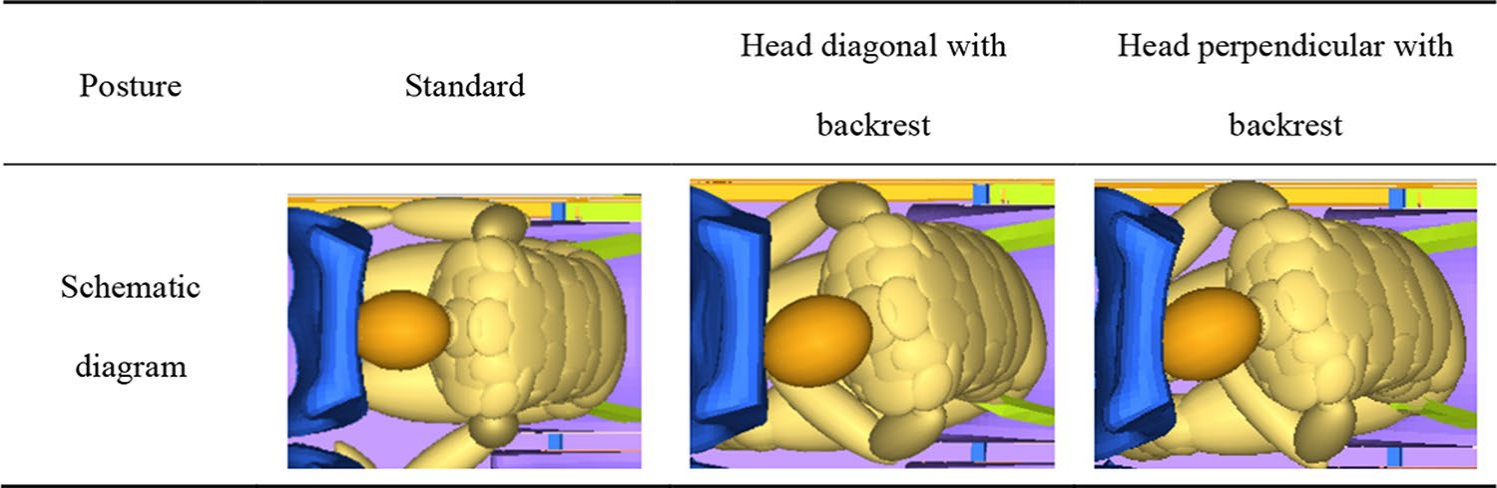
The head impact position with the front seat when γ is 30°.

**Fig. 9:**
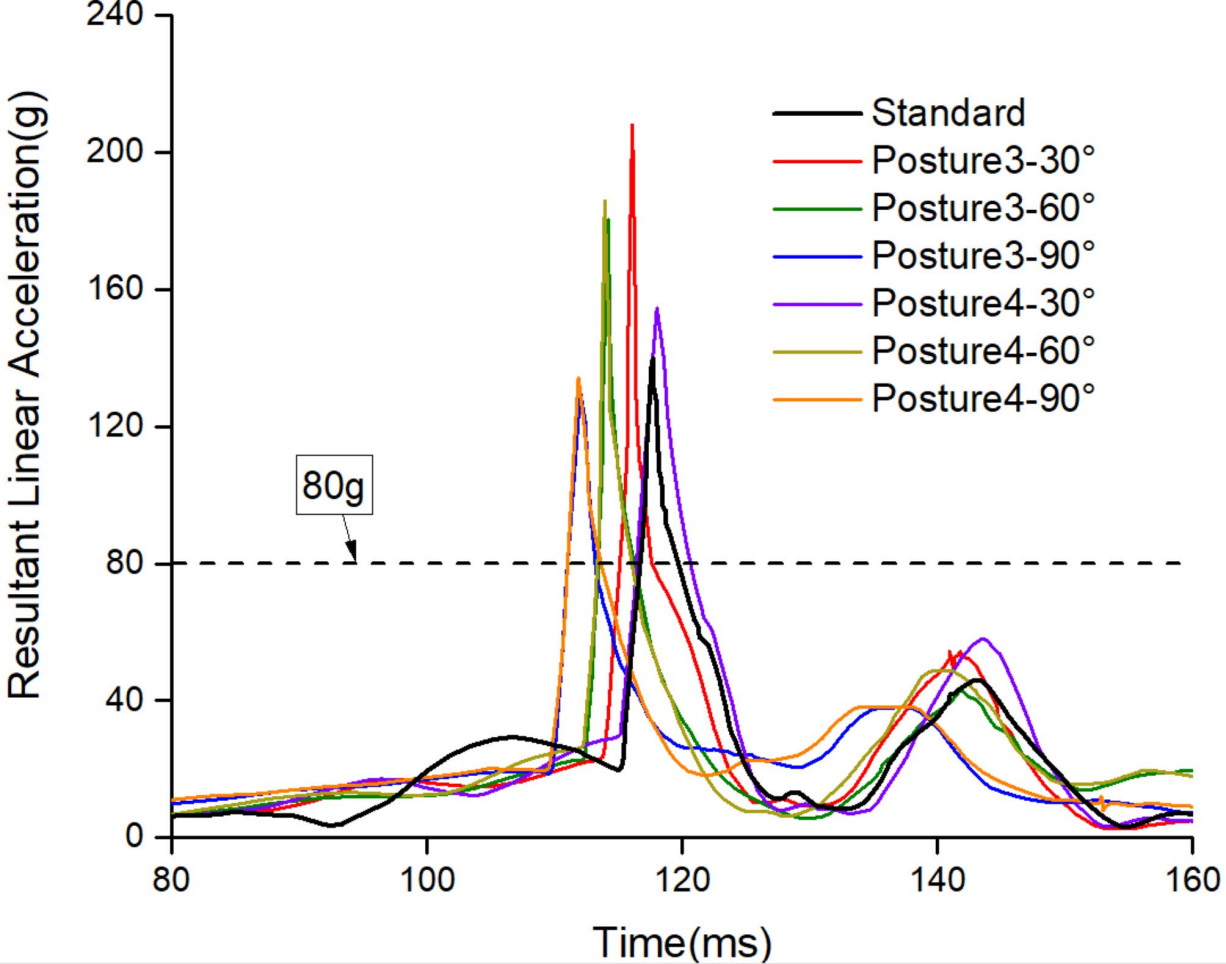
The resultant linear acceleration between 80ms and 160ms for the turned torso posture.

Under different angles in the turned torso posture, the occupant’s head acceleration curves vary, as seen in Fig. 9. The time of peak acceleration changes with collision time. Among these, the maximum peak acceleration is 208 g for the head diagonal with backrest posture when γ is 30°.

#### Turned torso-head diagonal with backrest

**Fig. 10:**
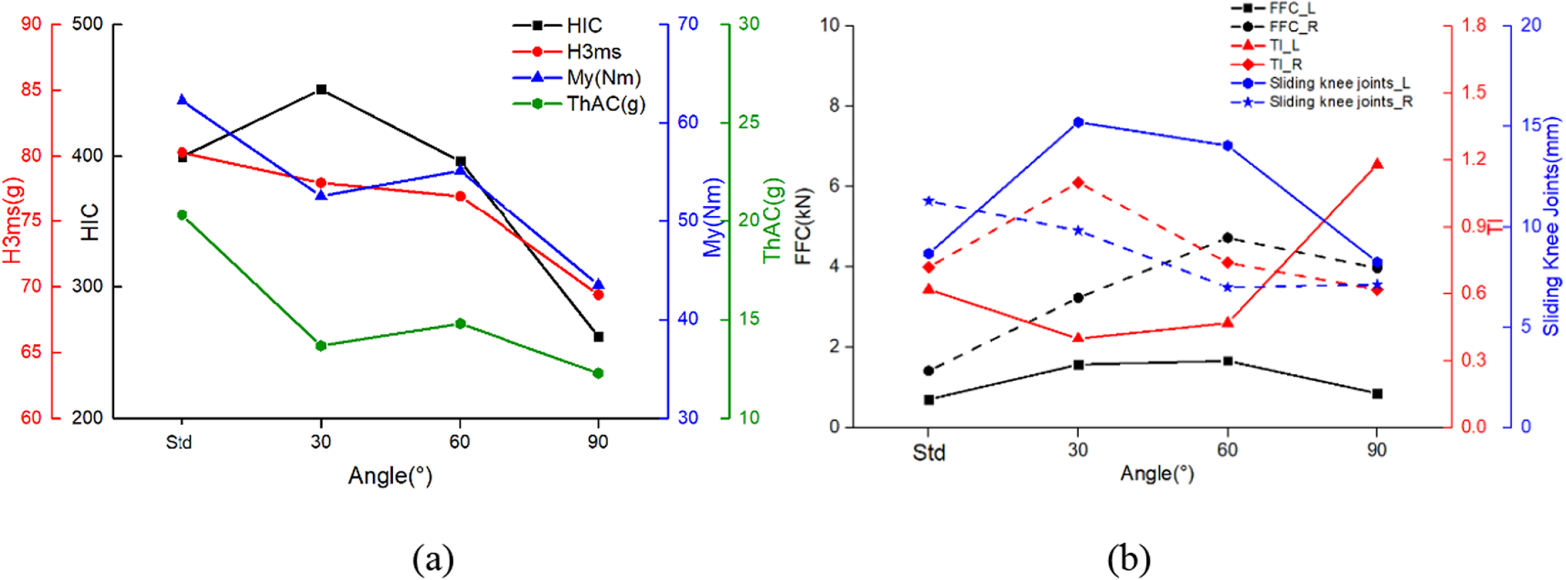
The comparison of occupant injuries for (**a**) head, neck, thorax, (**b**) femur, tibia and knee between different turned torso- head diagonal with backrest posture.

As shown in Fig. 10, the larger the value of γ, the smaller the values of HIC and H3ms for the occupant’s head. At γ of 90°, the HIC is only 262.1 and the H3ms is 69.40 Nm, both of which are much smaller than the injury values in the standard posture. Compared to the standard posture, the injury values of turned torso occupant have a greater reduction in both My and ThAC, with maximum reductions of 30.18% and 39.49%, respectively. As seen the kinematic response in Table 4 and the injury values in Fig. 10b, the turned torso results in a higher FFC for the right femur compared to the left, with both femurs experiencing higher loads than in the standard posture. From the TI values, it is evident that as γ increases during the collision process, the damage to the right tibia decreases while that of the left tibia increases. The slip conditions of the knees on both sides exhibit a similar decreasing trend. Furthermore, when γ is 30°, the sliding of the left knee joint exceeds the limit of 15 mm. The reason for this is that the occupant’s left lower limb impacts the front seat for the longest duration.

#### Turned torso-head perpendicular with backrest

**Fig. 11:**
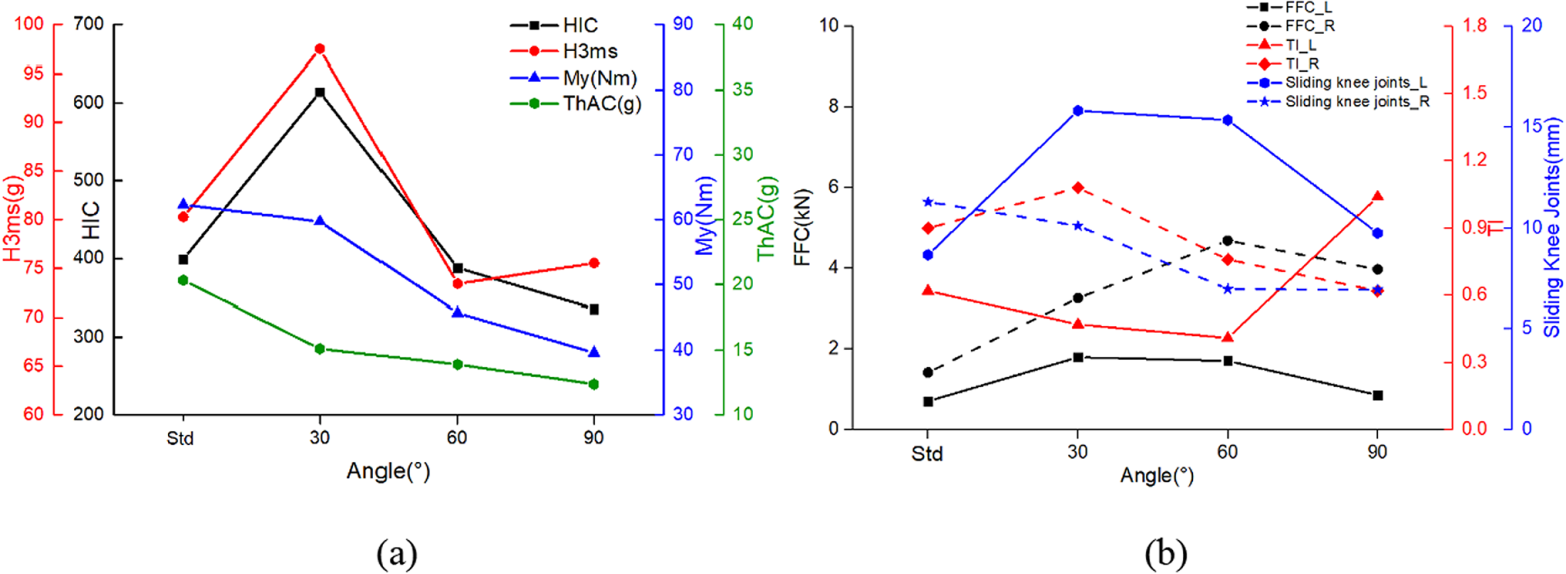
The comparison of occupant injuries for (**a**) head, neck, thorax, (**b**) femur, tibia and knee between different turned torso-head perpendicular with backrest posture.

Due to differences in the deflection of dummy head, there are variations in injury values compared to the head diagonal with backrest posture, showing in Fig. 11. Similarly, when γ is 30°, both postures result in the most severe injuries to the occupants’ heads. Furthermore, under the head perpendicular with backrest posture, HIC and H3ms are more severe, reaching 614.16 and 97.49 g respectively, exceeding the limits of 500 and 80 g. The difference arises at γ is 60°, where the head perpendicular with backrest posture is slightly safer, while at γ is 90°, the head diagonal with backrest posture is safer. Both neck and chest injuries decrease with increasing angles. The trends of values in FFC, TI, and knee sliding displacement are consistent for both postures. Although the numerical differences are small, at γ is 90°, the difference in the left knee sliding displacement is most significant, reaching 18.18%.

## Discussion

In coaches, occupants’ postures vary unpredictably. Especially during long-distance travel and night travel, occupants tend to adopt various sleeping postures in fatigued seating environments. One significant characteristic of the sleeping posture is that the occupant’s head is resting on the seat backrest, which may not necessarily be in the central position. This directly affects the timing and position of head and front seat collisions during coach collisions, thereby influencing injuries to the head, neck, thorax, and lower limbs of the occupants. However, in regulatory tests for coaches, such as ECE R80 and GB13057 ^20^, the dummy postures specified for safety experiments are all fixed positions. Comparisons of the injury values between standard and sleeping postures reveals that some sleeping postures result in significantly severe injuries, such as head injury in the turned torso posture. This highlights that standard postures only ensure occupant safety to a certain extent and may not be representative of real-world scenarios.

In this study an interrelated was found between body parts in different sleeping postures of the occupants. Turned torso sleeping posture illustrates this. From the head tilted sideway posture, it was found that head injuries are exacerbated with head deflection. At the same time, in the slide down on seat in neutral position posture, extending the time of knee collision with the front seat can reduce head injuries for occupants. Due to the correlation between body parts in the turned torso sleeping posture, the transformation involves variations in the head, arms, torso, and legs. The combined effect of these factors results in varying impacts on the head for occupants under different γ angles (Fig. 11a). When γ is 30°, the HIC is greater than the standard posture. And at other angles, it is lower than the standard posture. Anton et al.^12^ also confirmed in their study that random occupant postures have a significant effect on occupants, and these injury values are dispersive. Therefore, in the evaluation of occupant safety, controlling a single parameter change may not guarantee occupant safety; instead, a comprehensive consideration of multiple factors is necessary.

For occupant head injuries, both HIC and H3ms need to be considered. When α is at 20° in head tilted sideway posture, the HIC value is 480.1, which is below the requirements of ECE R80. However, the H3ms dramatically increases to 88.53 g, which is more than 80 g. The reason for this is shown in Fig. 6b, although the peak resultant linear acceleration is not the highest when α is 20°, the acceleration with a duration exceeding 3ms is the highest.

In this study, it was observed that the neck was not adequately protected. Although the turned torso posture significantly improved the neck injury for occupants, subsequent simulations of different sleeping postures revealed that in half of the cases, neck injuries exceeded the limit value of 57Nm in ECE R94. This was also evident in the sled test of real experiments, where the My value reached 71.8Nm. Qian Peng et al.^7,8^ also found issues with neck My exceeding the limit during frontal collisions in coaches. However, the criteria for the neck in regulations are not comprehensive. For instance, ECE R80 does not include criteria for neck.

**Fig. 12:**
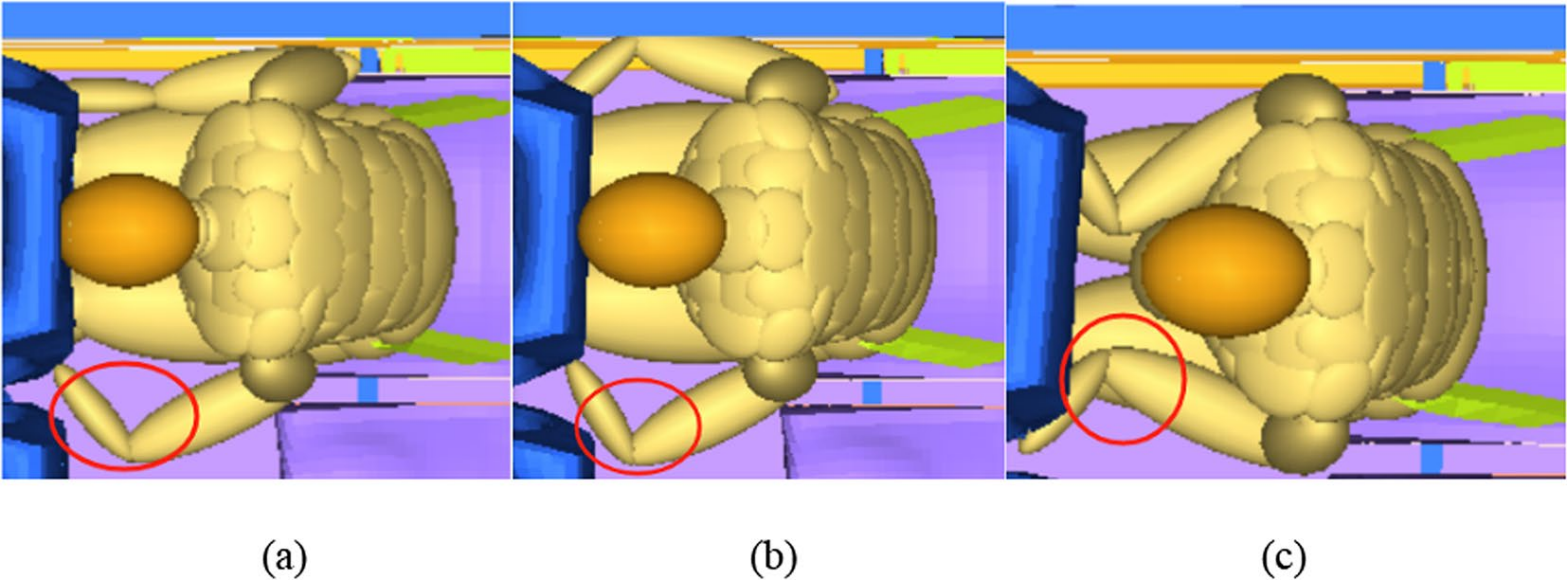
Different changes of the occupant’s arms at (**a**) 5°, (**b**) 10° and (**c**) 15°ofβ.

In the collision, the change in the position of the occupant’s arm may affect the injuries of upper body. When β is 15°, the trend of the occupant’s injury values changes compared to other angles. H3ms and ThAC show an upward trend, while My shows a downward trend. The possible reason is shown in Fig. 12. when β is 5° and 10°, the wrists are folded outward, the elbows are extended outward, and the arms exert no force on the front seat. In contrast, when β is 15°, the wrists are bent inward, the elbows are inward, and the arm has a thrust against the front seat. This changes the variation of the occupant’s injury values. Zhang Xiaolong et al. also found that the support of the arms may result in the increase of the thorax acceleration^21^.

The simulation results for various sleeping postures show that when occupants’ femurs bear a significant portion of the impact force during collisions, it is advantageous for reducing injuries to the occupant body parts. Slide down on seat in neutral position posture is the typical situation. Due to the proximity of occupants to the front seats, they endure the force for a longer duration, which disperses the impact force across all the body. As a result, this reduces the injury values for the head, thorax, tibia and knee of the occupants. The same situation occurs turned torso posture. Leledakis et al.^11^ in their study also confirmed that postural changes in the lower limbs have the greatest effect on the whole body of the occupant.

The knee sliding displacement of the occupant is not only related to the force borne by the femur, but also to the stiffness of the object struck by the knee. As shown in Table 8, in the turned torso posture, the right leg impacts the foam of the seat, while the left leg impacts on the frame of the seat. The occupant’s tibia cannot have a significant displacement after impacting the high stiffness frame, while the femur continues to move forward. This results in a larger displacement of the tibia relative to the femur, which is knee sliding displacement. This is also the reason why there is a big difference in the knee sliding displacement between the left and right knees of the occupant.

**Table 8:**
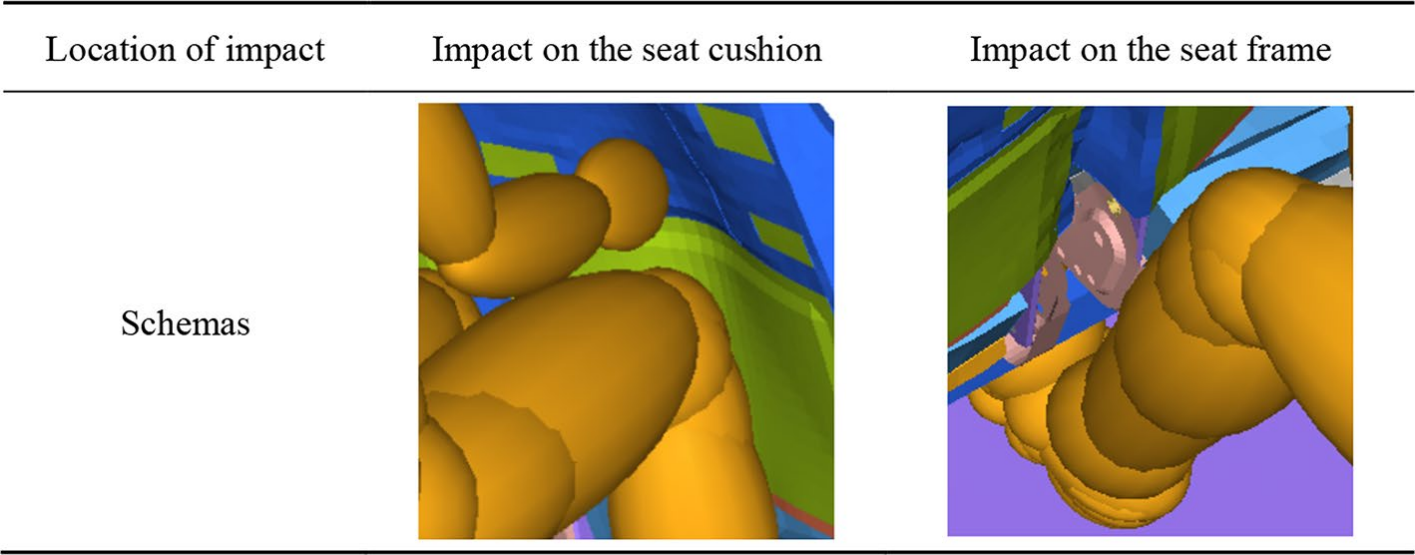
Schematic schemas of the location of lower limb impingement.

In four sleeping postures, when β is 15° in slide down on seat in neutral position posture, γ are 60° and 90° in turned torso-head diagonal with backrest posture, and γ is 90° in turned torso-head perpendicular with backrest, all injury values meet the requirements of ECE R80 and ECE R94. Among them, the sleeping posture of lowest risk is turned torso-head diagonal with backrest sleeping position when γ is 90°, where the values for head, neck, and chest injuries are the lowest. In contrast, turned torso-head perpendicular with backrest at γ of 30° is the highest risk sleeping posture with head, neck and knee injury values exceeding the limits. The highest risk sleeping posture-turned torso posture-also reflects that previous research, which focused only on random seating postures based on the standard posture, does not fully represent the generality of occupant injuries.

There are some limitations in this study. The research methodology is based on ECE R80 sled test, which has some differences in terms of the test requirements such as crash acceleration curve. Regulations such as FMVSS 208 and real crash acceleration curves^17^ are different from the curves specified here, which will affect the results of the study. Additionally, occupants of different sizes exhibit significant differences in dynamic responses and injury outcomes in coach crashes, such as small-sized female dummy.

This study contributes to the research on the safety of occupants in different sleeping postures in coaches. The effects of different sleeping postures show the importance of developing protection systems and improving evaluation systems. Thinking about sleeping postures where injury values exceed the standard posture, a more sophisticated evaluation system could help improve occupant safety in the real world.

## Conclusion

To study the effect of sleeping posture on the occupant’s injury, the dummy was set to sleep at different angles to obtain injury values. Comparing the injury conditions with those in the standard posture, it is observed that different sleeping postures have varying impacts on occupants’ injuries. Firstly, four sleeping postures result in a reduction in thorax injuries for the occupants by up to 39.49% compared to the standard posture of ThAC. In the head tilted sideway posture, the deviation angle of the head exacerbated the head injuries of the occupants. Compared to the standard posture, the HIC was elevated by 23.87% and the H3ms by 10.29%. Occupants in the slide down on seat in neutral position posture are cushioned by their knees, reducing their head injuries. The turned torso posture is beneficial in reducing the neck moment.

Among the above four sleeping postures, the turned torso-head diagonal with backrest posture with a γ of 90° is the lowest risk, while the turned torso-head perpendicular with backrest posture with a γ of 30° is the highest risk.

The study of these sleeping postures is helpful in identifying representative dummy posture in the test, which can improve occupant safety in frontal collisions of coaches. Simultaneously, this research also contributes to advancing the design of coach structure and the implementation of test at enhancing occupant safety.

## Data Availability

All data generated or analysed during this study are included in this published article.
